# The Immunomodulatory Potential of Short-Chain Fatty Acids in Multiple Sclerosis

**DOI:** 10.3390/ijms25063198

**Published:** 2024-03-11

**Authors:** Laura Barcutean, Smaranda Maier, Mihai Burai-Patrascu, Lenard Farczadi, Rodica Balasa

**Affiliations:** 1 Neurology Department, University of Medicine, Pharmacy, Science and Technology “George Emil Palade” Targu Mures, 540142 Targu Mures, Mures, Romania; laurabarcutean@gmail.com (L.B.); rodica.balasa@umfst.ro (R.B.); 2 Molecular Forecaster Inc., Montreal, QC H3A 2L1, Canada; mihai.buraipatrascu@molecularforecaster.com; 3 Center for Advanced Medical and Pharmaceutical Research, University of Medicine, Pharmacy, Science, and Technology “George Emil Palade” Targu Mures, 540142 Targu Mures, Mures, Romania; lenard.farczadi@umfst.ro

**Keywords:** multiple sclerosis, butyrate, propionate, acetate, valerate, short-chain fatty acids, microbiota, microbiome

## Abstract

Multiple sclerosis (MS) is a chronic inflammatory and neurodegenerative central nervous system (CNS) disorder, characterized by focal inflammation, demyelination, irreversible axonal loss and neurodegeneration. The proposed mechanism involves auto-reactive T lymphocytes crossing the blood–brain barrier (BBB), contributing to inflammation and demyelination. Pro-inflammatory Th1 and Th17 lymphocytes are pivotal in MS pathogenesis, highlighting an imbalanced interaction with regulatory T cells. Dysbiosis in the gut microbiota, characterized by microbial imbalance is implicated in systemic inflammation, yet its exact role in MS remains elusive. Short-chain fatty acids (SCFAs), including valerate, butyrate, propionate, and acetate, produced through dietary fiber fermentation by the gut microbiota, modulate inflammation and immune responses. Particularly, butyrate and propionate exhibit pronounced anti-inflammatory effects in both the gut and CNS. These SCFAs influence regulatory T lymphocyte expression and BBB permeability. This review discusses the potential therapeutic implications of SCFA in MS, highlighting their ability to modulate the gut–brain axis and restore immune balance.

## 1. Introduction

Multiple Sclerosis (MS) is a chronic inflammatory and neurodegenerative disorder affecting the central nervous system (CNS), characterized by focal inflammation and demyelination followed by irreversible axonal loss and neurodegeneration [1,2,3]. One of the proposed mechanisms for inflammation and demyelination is the “outside-in” theory, where auto-reactive T lymphocytes from the periphery cross the blood–brain barrier (BBB) and penetrate into the CNS where they exert inflammation, leading to demyelination [4]. CD4+Th lymphocytes regulate adaptive immunity by secreting pro and anti-inflammatory cytokines. Naïve CD4+Th cells become activated in the peripheral lymph nodes and differentiate under the influence of various polarizing cytokines, leading to the development of both pro-inflammatory and anti-inflammatory phenotypes. Pro-inflammatory Th1 and Th17 lymphocytes play a pivotal role in the development, onset, and progression of MS [5]. The accepted theory regarding the pathogenesis of MS suggests an imbalanced interaction between pro-inflammatory, effector T cells (Teff) and lymphocytes with the capacity to regulate the function of reactive T cells, the regulatory T cells (Treg) [6]. Additionally, B lymphocytes contribute to the disease pathogenesis by antibody secretion, antigen presentation to T lymphocytes and the production of pro-inflammatory cytokines [7].

While the exact etiopathogenesis of MS remains elusive, factors contributing to systemic inflammation, including dysbiosis of the gut microbiota, have been postulated [8].

**Figure 1:**
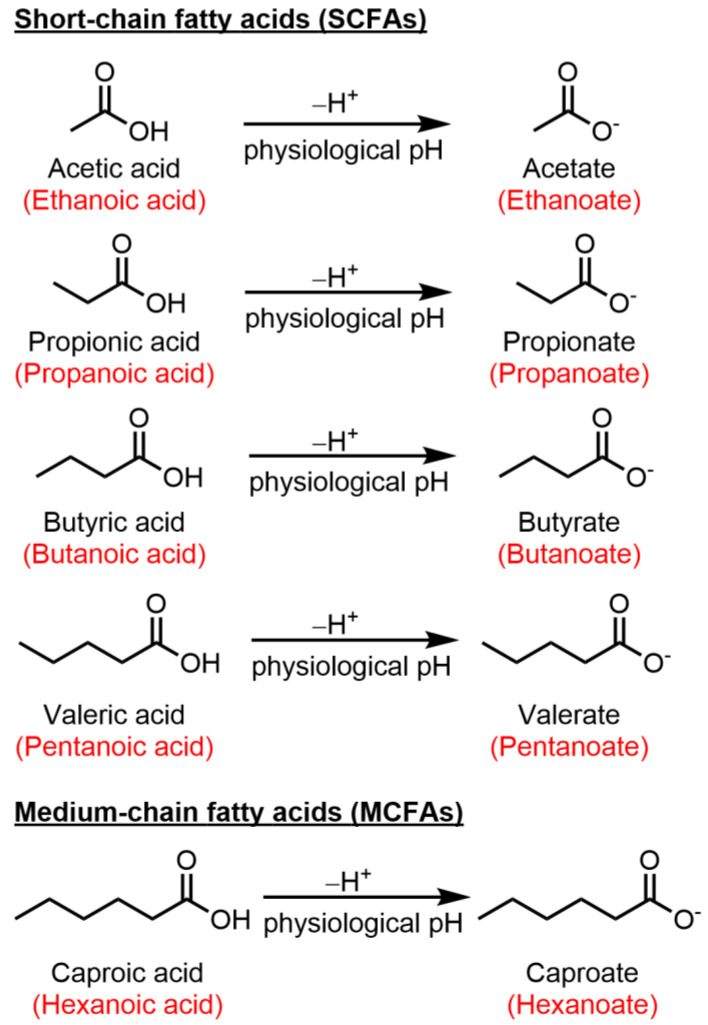
Chemical structures of key SCFAs and caproic acid, and their conjugate base form. Common names given in black, International Union of Pure and Applied Chemistry (IUPAC) naming in red.

The gut–brain axis serves as a dynamic communication system bridging the gap between the gastrointestinal tract and the CNS. Within this intricate network, there exists a complex interplay involving the enteric nervous system, neural pathways, immune cells, and the gut microbiota. Essentially, at its core, the gut–brain axis functions as a two-way signaling pathway through which the enteric system exerts influence on the brain, and conversely, the brain affects the enteric system [9]. The key element of this ecosystem is the intestinal microbiome, which consists of a diverse array of microorganisms like bacteria, yeast, and viruses. The dominant microorganisms are by far bacteria, of which the two most important phylae are (1) *Firmicutes* and (2) *Bacteroidetes* [10]. However, the composition of gut microbiota exhibits substantial variability within the same individual, across different locations within the gastrointestinal tract, and this variability can dramatically fluctuate from one person to another [11]. The by-products produced by the bacteria in the two phylae include short-chain fatty acids (SCFA), for which there is a growing body of evidence showing their involvement in modulating immune responses by promoting the balance between Treg and Teff [12,13,14]. Representative SCFAs include acetic, propionic, butyric, and valeric acid. At physiological pH, SCFAs are found in their conjugate base form: acetate, propionate, butyrate, and valerate (Figure 1).

When dysbiosis, a state characterized by microbial imbalance, disrupts this finely tuned equilibrium, it sets off a cascade of events leading to systemic inflammation. This, in turn, directly impacts the inflammatory processes that are intimately linked with conditions such as MS. This narrative review provides a comprehensive analysis of the potential immunomodulatory roles of SCFA and their impact on inflammation and the CNS in MS. We conducted an extensive literature search up to November 2023 using PubMed, Scopus, and Google Scholar. The search included the following terms: “short-chain fatty acids”, “butyrate/butyric acid”, “propionate/propionic acid”, “acetate/acetic acid”, “valerate/valeric acid”, “gut microbiota/microbiome”, and “multiple sclerosis”. We included both past reviews and original articles to ensure topic relevance.

## 2. Short-Chain Fatty Acids

SCFAs are organic compounds that are produced through the anaerobic fermentation of dietary fibers by beneficial microorganisms residing in the gastrointestinal tract, commonly referred to as the gut microbiota. These fatty acids are characterized by having a relatively small number of carbon atoms (2–5) in their molecular structure [15]. Acetate (C2), propionate (C3), butyrate (C4) and valerate (C5) (Figure 1) collectively play complex roles between gut microbiota and host health. After intestinal absorption, the SCFAs cross into the systemic circulation and exert their effects both at a cellular and molecular level [16]. Unlike SCFA, medium-chain fatty acids are sourced directly from the diet and are generated by the liver through peroxisomal beta-oxidation of long-chain fatty acids. Caproic acid (C6), a medium-chain fatty acid has been shown to induce the differentiation of Th1 and Th17 lymphocytes [17,18]. An overview of the functions of caproic action falls outside the scope of this review and has been briefly mentioned in Figure 1 and Table 1. Numerous studies attest to a reduction in SCFA levels in MS patients compared to healthy individuals. A summary of these studies is represented in Table 1.

### 2.1. Butyric Acid (Butyrate)

Butyrate is the most extensively studied SCFA in the human microbiome. It occupies a prominent position due to its dual capacity to regulate inflammation in two crucial compartments: the periphery (intestinal compartment) and the CNS compartment. In the gut, butyrate contributes significantly to inflammation reduction, leading to a global decrease in inflammation. Moreover, in the CNS, its demonstrated ability to traverse the BBB enables it to modulate its permeability, influencing the passage of inflammatory cells [19,32].

**Figure 2:**
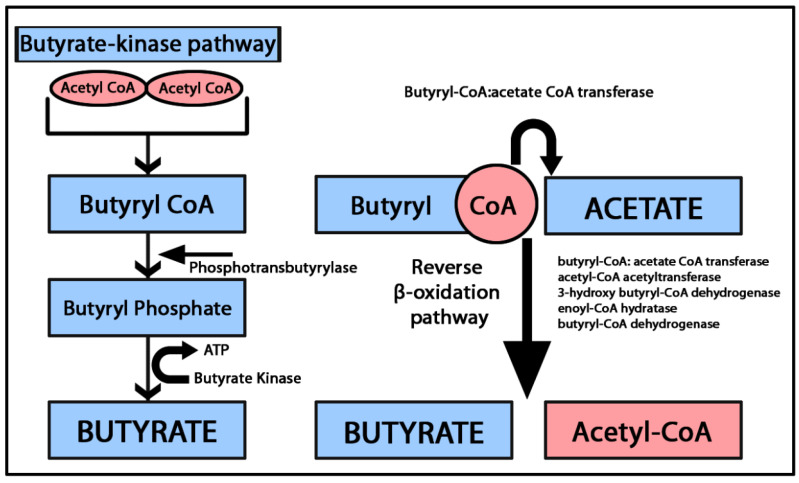
Graphical representation of the butyrate synthesis pathways. In the butyrate kinase pathway, two molecules of acetyl CoA combine to form butyryl CoA. Subsequently, phosphotransbutyrylase catalyzes the conversion of butyryl CoA into butyryl phosphate. Finally, butyrate is generated from butyryl phosphate through the activity of butyrate kinase. The butyryl-CoA: acetate CoA-transferase pathway converts butyryl CoA and acetate by a series of β oxidation enzymatic reactions into butyrate and acetyl CoA [34,35].

The primary sources of butyrate include the fermentation of non-digestible fibers in the colon and butyrate-producing bacterial genera, such as *Clostridium* and *Eubacterium* [33]. Butyrate-producing bacteria follow two main pathways: the (1) butyrate kinase pathway, which converts butyryl-coenzyme A (CoA) into butyrate and (2) butyryl-CoA: acetate CoA-transferase pathway, where butyryl-CoA is converted to butyrate through a series of enzymatic reactions involving β-oxidation mediated by butyryl-CoA: acetate CoA transferase, acetyl-CoA acetyltransferase, 3-hydroxy butyryl-CoA dehydrogenase, enoyl-CoA hydratase and butyryl-CoA dehydrogenase [34,35]. The butyryl-CoA:acetate CoA transferase is the most important coenzyme implicated in butyrate synthesis due to its role in transferring the CoA from the butyryl-CoA to acetate, forming butyrate and acetyl-CoA [34]. An overview of the butyrate-producing pathways is presented in Figure 2.

Butyrate serves as the primary energy source for colonocytes and is a significant regulator of gut immunity, notably by upregulating the expression of Treg cells [36]. Furusawa et al. demonstrated that butyrate acts as a Foxp3+ promoter that subsequently induces CD4+ T cell differentiation to a Treg lineage [37]. Additionally, *Bacteroides fragilis* a species within the *Bacteroidetes* class, known for producing endogenous IL-10 Foxp3+ Treg, also produces butyrate [38,39]. A recent meta-analysis has provided compelling evidence indicating a noteworthy shift in the microbial landscape of individuals affected by MS, specifically a decrease in *Firmicutes* phylum and an increase in *Bacteroidetes* phylum [9]. Both are crucial contributors to SCFA production. Studies consistently demonstrate that MS patients have insufficient butyrate levels and altered gut-microbiota composition, lacking SCFA-producing bacteria [19,40,41]. The Multiple Sclerosis Microbiome Study reveals a significant decrease *in Faecalibacterium prausnitzii* (*F. prausnitzii*) of the *Firmicutes phylum* in untreated MS patients, compared to those treated with interferon-beta. *F. prausnitzii*, an important butyrate producer, also exhibits anti-inflammatory properties, including inhibiting IL-8 production, upregulating Tregs, and blocking expression via the nuclear factor κB [42]. Levi et al., in a study involving fecal stool samples, observed that MS patients typically exhibit a depletion of butyrate-producing bacteria. However, in their study, no differences in serum levels of butyrate were found [19]. 

#### 2.1.1. Butyrate and Blood-Brain Barrier

Butyrate’s pivotal role in maintaining tissue homeostasis extends to enhancing the integrity of both the intestinal barrier and BBB. One of its crucial functions is to increase the transendothelial electrical resistance (TEER) in the intestinal barrier by activating the AMP-activated protein kinase (AMPK) [43,44]. AMPK upregulates the assembly of tight junctions (TJ), thereby ensuring the integrity of one of the most exposed barriers in the organism. In models of Caco-2 intestinal cells, butyrate has been shown to enhance the intestinal barrier function through AMPK activation. Higher levels of butyrate were associated with a reduction in ATP-dependent AMPK phosphorylation [45]. Low intracellular ATP levels corresponded to low butyrate levels, whereas higher levels of butyrate supported the synthesis of TJs Furthermore, butyrate increases the expression of claudin-3 and -4 on intestinal endothelial surfaces threatened by LPS, indicating a selective regulation of TJ proteins [45,46].

In terms of defense, both the intestinal barrier and the BBB have evolved in response to maintaining tissue homeostasis. They consist of a complex cellular layer serving as a physical barrier, sensitive to a variety of immune cells [47]. The BBB represents a complex functional assembly of endothelial cells, TJs, and supportive cells such as pericytes and astrocytes [46]. In MS, a key characteristic is the disruption of the BBB, allowing cells to enter the brain parenchyma. The direct assessment of the BBB can be challenging due to its inaccessibility and inherent dynamic nature. Although imaging techniques and laboratory assays, such as cerebrospinal fluid evaluation, allow for an indirect BBB assessment, their utility is constrained. One of the methods used to investigate BBB permeability in vitro is TEER, by impedance spectroscopy [48], which allows the monitoring of the electrical resistance of the endothelial tissues in a non-destructive manner. Several in vitro studies demonstrated that butyrate increases TEER and acts as a ubiquitous barrier protector [32,49]. In in vivo studies of stroke murine models, butyrate supplementation reduced BBB permeability and enhanced the expression of claudin-5 and zonula occludens-1 [50,51]. Similar findings were reported for mice models of Parkinson’s disease, traumatic brain injury and sepsis [52,53,54]. Despite the challenges in direct BBB assessment, techniques like TEER have shown that butyrate enhances barrier integrity, reducing permeability and promoting the expression of tight junction proteins.

Butyrate possesses the capability to traverse the BBB, facilitated by the presence of monocarboxylate transporters (MCTs) that are notably abundant in endothelial cells at the BBB level and in certain CNS cells [55]. Some MCTs are synthetized by intestinal cells, such as MCT-1 and MCT-2 [56]. MCT-1 is primarily expressed in the apical membrane of human intestinal epithelial cells, while in the CNS it is widely found on oligodendrocytes, astrocytes and neurons [57,58,59]. Butyrate, together with propionate and acetate, serves as an energy source for MCT-1 in a sodium-dependent manner [60,61]. Aside from butyrate transport, MCT-1 acts as a relay for lactate transport from the astrocytes to oligodendrocytes and neurons [57,58]. Long-term exposure to butyrate promotes MCT-1 expression via NF-κB while increasing the stability of MCT-1 gene regulation via microRNA [59,62,63]. Furthermore, MCT-1 supports oligodendrocyte survival [64], promotes lactate metabolism from oligodendrocyte to the axon, and encourages axonal plasticity [65,66]. MCT-2 is mainly expressed by neurons [67] and exclusively transports butyrate. In murine studies, researchers demonstrated that MCT-2 is expressed in the hippocampus and the cerebellum, and it plays an important role in synapse repair, particularly within the postsynaptic density [60]. This offers potential avenues for therapies that can directly target the CNS. In cerebral ischemia studies, upregulation of MCT-1 and -2 was discovered to maintain the balance of lactate among oligodendrocytes, astrocytes, and neurons [57].

#### 2.1.2. Butyrate and Immunomodulation

Butyrate interacts with G protein-coupled receptors (GPCRs) located on the cell surface, mainly GPR41 and GPR109A [68]. Upon butyrate activation, these GPCRs trigger various intracellular pathways involved primarily in immune modulation. GPR41 is the most abundant and is present in peripheral blood mononuclear cells, such as monocytes and dendritic cells. The up-regulation of GPR41 plays a crucial role in mediating the sympathetic nervous system, contributing to metabolic homeostasis. Additionally, the regulation of GPR109A by butyrate indirectly induces Treg expression and interleukin (IL)-10 secretion [69,70,71]. T lymphocytes lack the GPCRs that bind to SCFAs, thus the SCFAs–T lymphocytes modulation is most likely triggered by histone deacetylase (HDAC) inhibition [72]. Therefore, one of the suggested immunomodulatory roles attributed to butyrate is the capacity to inhibit HDACs. This mechanism was demonstrated by Chen et al. in a murine MS cuprizone model where the authors explored the effects of SCFA (particularly butyrate) on cuprizone-induced demyelination. Their findings demonstrated that butyrate treatment substantially improved demyelination, while acetate and propionate showed limited or no effects. Although no significant changes were observed in microglial activation or plasticity with SCFA treatment, the supplementation of butyrate was linked with oligodendrocyte maturation, which likely contributed to the process of remyelination. Furthermore, the authors compared butyrate with a HDAC (see below for description) inhibitor, trichostatin A. Both butyrate and trichostatin A supplementation significantly enhanced remyelination, suggesting that butyrate influences oligodendrocytes acting as an HDAC inhibitor [73].

**Figure 3:**
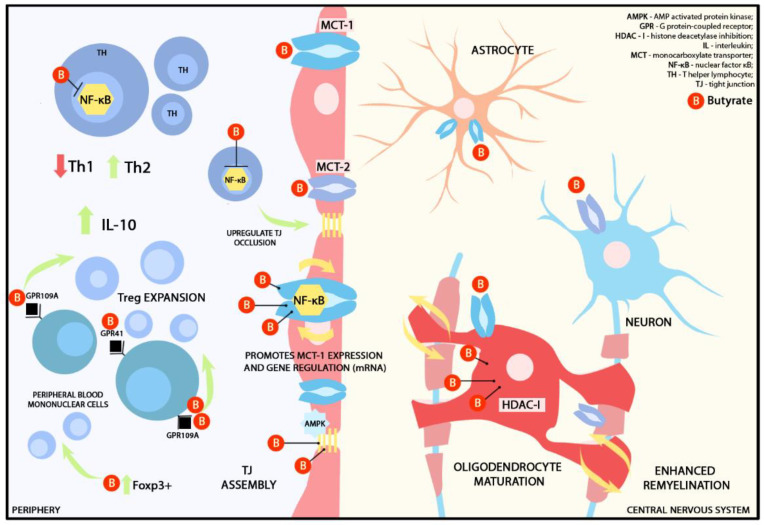
Graphical representation of presumed immunomodulatory effects of butyrate in the periphery and the CNS. MCT-1 receptors are widely distributed across the BBB and CNS cells, such as astrocytes, neurons, and oligodendrocytes, while MCT-2 is primarily located on neurons [56]. MCT-2 exclusively transports butyrate. Butyrate functions as an energy source for MCT-1 in a sodium-dependent manner. Prolonged exposure to butyrate promotes MCT-1 expression via NF-kB and enhances the stability of MCT-1 gene regulation through mRNA [43,44]. Additionally, butyrate acts as HDAC-I on oligodendrocytes, promoting remyelination. At the BBB level, butyrate activates AMPK, which, in turn, upregulates the assembly of TJ. In the periphery, butyrate acts as a Foxp3+ promoter that subsequently induces CD4+ T cell differentiation to a Treg lineage. It also interacts with GPCRs, such as GPR41 and GPR109A, found on the surface of peripheral blood mononuclear cells. Upregulation of GPR109A induces Treg expression and IL-10 secretion. T lymphocytes lack GPCRs that bind to butyrate or any other SCFA; therefore, T lymphocyte modulation is triggered by HDAC-I. Butyrate also supports the synthesis and assembly of TJ [43,69,70].

HDACs represent a class of enzymes critically involved in the epigenetic regulation of chromatin and gene expression [74]. HDAC inhibitors (HDAC-i) have been extensively studied for their potential in suppressing malignant cells, particularly in the context of glioblastoma [75,76]. Bae et al., in a murine experimental autoimmune encephalomyelitis (EAE) model, demonstrated that CDK-506, an HDAC6-i decreased T cell and macrophage passage in the spinal cord, reduced Th1 cell-related cytokines and improved the BBB integrity by upregulating occludin expression [77]. It also indirectly up-regulated Th2-dependent immune responses. This effect is achieved by inhibiting the activation of NF-κB, a key transcription factor responsible for regulating the production of inflammatory cytokines. The suppression of NF-κB, in turn, encourages the production of IL-10 [78], a pivotal anti-inflammatory cytokine with significant immunomodulatory functions. In the context of MS, this is particularly relevant, as it can help counterbalance the pro-inflammatory responses that contribute to the pathogenesis of the disease. This could provide an explanation for the hypothesis that butyrate (and to a lesser extent, propionate) may have a role in preventing relapses in patients with MS [8]. The HDAC-i role was also demonstrated in ex-vivo microglia from fiber-fed mice. Microglial cultures were treated with a mixture of sodium butyrate and acetate leading to a significant decrease in HDAC activity, NF-κB translocation and TNF alfa expression after lipopolysaccharide (LPS) administration. This suggests that SCFA inhibits inflammatory responses not only in the periphery and at the level of the BBB but also beyond [79]. The major functions of butyrate in the periphery and inside the CNS are represented in Figure 3.

No studies have been reported regarding the effects of direct supplementation of butyrate in human subjects. However, the beneficial effects of butyrate esters were demonstrated in a number of murine studies. The effects of methyl butyrate (MB), which is a methyl ester form of butyric acid routinely used as a food additive due to its fruity flavor, have been evaluated in the context of EAE suppression in animal studies. Animals treated with oral MB exhibited lower EAE severity scores compared to controls. Flow-cytometry analysis revealed lower CNS CD4+Th lymphocyte infiltrates in the treated group compared to controls. Furthermore, a reduction in the number of inflammatory cells (interferon (IFN)-γ+IL-17A-Th1 and IL-17A+IFN-γ-Th17) was noted. MB administration additionally prompted Treg expression in lymph nodes. In the periphery, as determined by immune cell function in the spleen, the same research group demonstrated that, following MB supplementation, no differences were found in the levels of pro-inflammatory cytokines (IFN-γ and IL-17), while the peripheral levels of IL-10 were significantly increased [80].

In a study by Saresella et al., an analysis of serum concentrations revealed that individuals with MS have reduced levels of butyrate but elevated levels of caproic acid. Caproic acid (Figure 1) is a medium-chain fatty acid directly derived from the diet. This contrast is particularly evident in their impact on T lymphocyte development: butyrate promotes the development of Tregs, while caproic acid enhances the differentiation of Th1 and Th17 cells. In MS patients, elevated caproic acid levels were positively associated with CD4+IFN-γ T lymphocytes, indicating their pro-inflammatory potential. Additionally, the authors established a connection between caproic acid levels and the permeability of the intestinal barrier via LPS expression, suggesting intestinal barrier damage in MS patients [20].

Spore-forming bacteria, including *Clostridia,* have been demonstrated to induce regulatory T-helper lymphocytes in the gut microbiome [54]. The *Clostridia* class belongs to one of the most abundant bacterial phyla in the gut, *Firmicutes* [81]. MS patients often exhibit a decrease in *Clostridia* clusters IV and XIVa, resulting in insufficient butyrate production [82]. In *ad libitum* fed EAE murine model, Calvo-Barreiro et al. demonstrated that EAE mice exposed to *Clostridia* strains harvested from healthy individuals’ stool samples exhibited a lower rate of demyelination in the spinal cord’s white matter compared to control mice. Although there were no significant differences in peripheral T-helper cytokine secretion between the two groups (including IFN-γ, IL-4, IL-10, and IL-17A), an increase in CD62L+Treg cells was observed, correlating with the observed clinical improvement, albeit not reaching statistical significance. The administered Clostridia strains have the capacity to stimulate butyrate production, leading to higher levels of serum butyrate compared to control mice. Furthermore, a positive impact on the motor function of EAE models was achieved by supplementing their diet with butyrate (one week before immunization and throughout the experiment). These findings suggest that oral treatment with Clostridia strains has the potential to enhance clinical outcomes in EAE and potentially restore gut dysbiosis in MS patients [83]. Although the therapeutic impact of butyrate alone was not as remarkable as that observed with Clostridia strains, the study underscores the promising translational implications of these results for individuals with MS.

While much research has focused on the role of T lymphocytes in MS, it is important to note the significant impact of SCFAs, particularly butyrate, on B lymphocytes as well. SCFAs enhance intracellular acetyl-CoA levels through acetyl/propionyl/butyril-CoA synthetase, a key enzyme in the mitochondrial Krebs cycle that is afterward integrated with the B lymphocytes energetic cycle [84,85,86]. This metabolic boost leads to the production of more energetically potent B cells, as demonstrated in murine studies [84]. Moreover, SCFAs play a regulatory role in B lymphocyte activity by promoting the upregulation of regulatory B cells, facilitating the secretion of IL-10, and stimulating T helper cells and follicular T helper cells.

### 2.2. Propionic Acid (Propionate)

Propionate is secreted in the gut by *Clostridia* and *Bacteroidetes* species and is commonly present in industrialized products like dairy and refined wheat, where it serves as a fungal inhibitor. Naturally occurring propionate in the gut possesses significant immunomodulatory properties, resembling butyrate, while elevated levels are linked to metabolic disorders such as mitochondrial dysfunctions [87,88,89].

Similar to butyrate, propionate exerts immunomodulatory functions via GPCRs such as GPR43, which is highly expressed in immune cells such as neutrophils, and the gut epithelium. The up-regulation of GPR43 has anti-inflammatory and anti-carcinogenic effects by promoting Treg differentiation [90]. To a lesser extent, it also binds to GPR41, additionally promoting hematopoiesis of dendritic cells (DCs) from bone marrow [91]. DCs play an important role in the pathophysiology of immune diseases such as MS due to their antigen-presenting properties to T lymphocytes [92]. In contrast to T lymphocytes that do not possess GPRs, DCs express GPR109a and GPR41. Nastasi et al. demonstrated that propionate and butyrate, but not acetate, reduce the expression of the pro-inflammatory cytokine IL-6 secondary to DC activation, with implications for shifting the immune response toward a regulatory phenotype. Furthermore, propionate reduces pro-inflammatory chemokine ligands such as CCL3, CCL5, CXCL9, and CXCL10, suggesting direct immunomodulatory effects on DCs [93].

Propionate supplementation in MS patients was linked to a shift in the immune system’s functionality toward a regulatory phenotype. Duscha et al. identified a significant reduction in stool and serum concentrations of propionate in human subjects, across all evaluated MS phenotypes (relapsing-remitting, secondary progressive and primary progressive) independent of the treatment. Furthermore, the administration of propionate at a daily dosage of 1000 mg was associated with an upregulation of Tregs facilitated by IL-10 secretion, along with a reduction in Th17 lymphocytes. Long-term propionate supplementation was associated with a reduced annual relapse rate and stabilized disease progression [28].

The complex immune regulation exhibited by propionate has been demonstrated in other studies, primarily indicating the overexpression of IL-10. Cavaglieri et al. assessed cytokine secretion in rat mesenteric lymph node lymphocytes and demonstrated that propionate supplementation increased IL-10 by 80% after 48 h [94].

In a recent study on MS patients aimed at examining the response of osteoporosis biomarkers to propionate supplementation, additional effects on Treg suppressive activities were demonstrated, particularly in relation to osteoclast differentiation [95]. Osteoporosis and bone fractures are prevalent among MS patients, primarily attributable to the ongoing requirement for corticosteroid therapy and the increased risk of falls associated with motor deficits and gait impairment throughout the course of the disease [96]. Supplementing the diet with propionate resulted in a 50% increase in peripheral Tregs and a 30% decrease in peripheral Th17 lymphocytes. Regarding bone biomarker turnover, the authors observed a noteworthy rise in serum osteocalcin and a reduction in β-CrossLaps, a biomarker indicative of bone resorption. Furthermore, there was a correlation between osteocalcin levels and peripheral Tregs, suggesting a potential positive impact on bone metabolism [95].

Haase et al. investigated the potential of propionate to counteract the detrimental effects of long-chain fatty acids during neuroinflammation. The mice were fed with mixtures of long-chain fatty acid lauric acid (LA) before inducing EAE. Afterward, they were treated with either water or propionate. The LA-fed mice exhibited higher EAE severity scores compared to controls but propionate treatment alleviated the clinical signs, bringing the disease course in line with the control group. Flow-cytometry analysis demonstrated that LA-fed mice exhibited high expressions of Th17 lymphocytes and decreased Tregs, but propionate treatment counteracted these effects [97]. These effects were also found in human studies. Zeng et al. demonstrated a strong correlation between fecal propionic and acetate levels and the expression of Treg cells, suggesting a promotion of regulatory differentiation in T lymphocytes [25]. Propionate has, therefore, shown promising results in mitigating the immune responses towards an anti-inflammatory state.

### 2.3. Acetate

Even though acetate has been the subject of fewer studies, it appears to be produced in the colon at three-fold higher quantities compared to butyrate and propionate. *Blautia hydrogenotrophica* and *Marvinbryantia formatexigens* from *Firmicutes* phylum are suggested to be the main producers in the human enteric system, by CHO fermentation [98,99]. Microbial fructose metabolism activates the acetate pathway for de novo lipogenesis. In terms of metabolic processes, a significant contrast between glucose and fructose metabolism lies in the rapid breakdown of high sugar consumption in the small intestine, while fructose consumption generates acetate through the gut microbiome [100]. Consequently, understanding acetate metabolism and its genesis hold potential significance for future therapeutic interventions in the field of autoimmune and metabolic diseases. Acetate can also be produced from acetyl-CoA, which is derived from glycolysis and must be coupled with CoA to engage in enzymatic reactions [101,102]. Liu et al. demonstrated that pyruvate dehydrogenase, along with reactive oxygen species such as hydrogen peroxide, can directly convert pyruvate into acetate [103].

Studies investigating acetate levels in MS have reported conflicting results. Pérez-Pérez et al., in a study involving 94 RRMS patients and 54 HC, analyzed acetate levels using liquid-chromatography mass spectrometry (LC-MS). The authors observed statistically higher levels of plasma acetate in MS patients compared to HC. Furthermore, when stratifying the MS cases based on disease severity using the Expanded Disability Status Scale (EDSS), patients with EDSS ≥ 5.0 had higher levels of acetate compared to those with EDSS ≤ 1.5. The calculated MS severity score also exhibited a positive correlation with acetate levels. Flow cytometry analysis demonstrated a direct correlation between acetate levels and IL-17-producing CD8+ T-helper lymphocytes [31]. Similar findings were documented by Cuello et al. in a study on pregnant MS patients, where acetate demonstrated a substantial increase during both pregnancy and the postpartum period compared to the HC group [21].

Conversely, among all the analyzed SCFA, only serum acetate levels were observed to be decreased in MS patients compared to HC, as reported in a recent study by Olsson et al. In the HC group, the authors documented negative correlations between serum acetate levels and interferon-γ, a significant pro-inflammatory cytokine with noteworthy implications as a mediator of inflammation in MS patients [30]. When assessing acetate levels in relation to disease activity, particularly in terms of the active brain lesions observed on magnetic resonance imaging scans, lower acetate levels were observed in individuals with inactive MS compared to HC [30]. This observation suggests a potential favorable association between acetate and disease activity in MS. In a study that examined plasma SCFA levels in SPMS patients, it was observed that acetate levels were decreased in comparison to control subjects. Furthermore, in the same study, acetate administration had a modest suppressive effect on EAE pathogenesis through an IL-10-mediated pathway [22].

In a mouse model of MS treated with methyl acetate (MA), an organic compound with a structural similarity to acetate primarily used in the cosmetic and textile industry, it was observed that the EAE score was reduced compared to mice treated with a control substance. Additionally, the levels of IFN-gamma and IL17A-secreting T-helper lymphocytes were lower than those in control mice. These findings indirectly suggested that the administration of MA reduced the infiltration of pro-inflammatory T-helper lymphocytes into the CNS. Importantly, it was confirmed that Th1 cells remained in the periphery due to an overexpression of Th1-associated chemokines, such as CXCR3, CXCL9, and CXCL10 [104]. This finding is crucial in terms of MS pathophysiology, where the peripheral activation of Th1 and Th17 lymphocytes is followed by BBB breakdown and subsequent CNS immune-mediated destruction. This finding holds significant implications for our understanding of MS pathophysiology. In MS, the peripheral activation of Th1 and Th17 lymphocytes is followed by BBB breakdown and the subsequent immune-mediated destruction in the CNS [105].

Despite ongoing research into its precise immunomodulatory functions, acetate’s fundamental role lies in stimulating the production of butyrate. This cross-feeding mechanism is demonstrated by *F. prausnitzii*, a dominant colon species, that relies on acetate for butyrate production [106,107]. In contrast, *Bifidobacterium adolescentis,* a prevalent Bifidobacterium species is involved in cross-feeding interactions with *F. prausnitzii*, influencing butyrate formation when provided with specific carbon sources like fructooligosaccharides or starch. These findings highlight the complex cross-feeding relationships among gut bacteria, influencing the production of beneficial metabolites like butyrate [108]. Given the ever-shifting and highly diverse nature of gut microbiota, contrasting results regarding the effect of acetate in MS patients and EAE models may be attributed to the pathway through which acetate is produced.

### 2.4. Valerate

The immunomodulatory effects of valerate, more commonly known as a branched SCFA have been less explored in the context of demyelinating conditions, such as MS. Lower concentrations of valerate are primarily produced by the intestinal microbiota through the fermentation of branched-chain amino acids. *Prevotella stercorea* and *Prevotella copri* of the *Bacteroidetes* class are the main producers of valerate in the gut lumen using amino-acid fermentation such as valine, leucine and isoleucine [109]. Additionally, some *Escherichia coli* species use thiolase to process valerate by butyryl-CoA and acetyl-CoA pathways [110,111]. *Mobilicoccus massiliensis*, a species within *Actinomycetota*, has been identified as a valerate-producing bacterium. In a study conducted by Yuille et al., the authors demonstrated that valerate exhibits concentration-dependent immunomodulatory properties similar to butyrate, functioning as a class I HDACi [111]. Class I HDACs play important roles in cell differentiation and survival and their inhibition carries important therapeutic avenues in neoplastic and inflammatory conditions [112,113].

In a study conducted by Luu et al., mice were fed with sodium butyrate or sodium valerate, and the researchers assessed the concentrations of SCFA and the immunological profile of B and T lymphocytes. The serum levels of all SCFAs were robustly expressed in wild-type mice but reduced in germ-free mice. Valerate administration inhibited the proliferation of Th17 lymphocytes, subsequently leading to a reduction in IL-17A secretion by down-regulating Th17-associated transcription factors such as STAT3, RORc, and TGFβ3 [114]. Moreover, in a subgroup of mice induced with EAE, valerate administration ameliorated EAE severity and reduced the number of infiltrating TCD4+ cells into the CNS. Additionally, the authors observed an increase in IL-10 expression. While no effects were noted on Tregs, valerate supplementation caused B lymphocytes to increase their IL-10 secretion. Notably, the same effect was not observed for butyrate supplementation, leading the authors to conclude that valerate, and not butyrate, may harbor significant therapeutic properties for enhancing the suppressive B lymphocyte phenotype [114]. Olsson et al. reported positive correlations between valerate and tumor necrosis factor and IFNγ, but after testing for multiple comparisons the results returned no statistical significance [30].

## 3. Conclusions

SCFAs play an important role in immune regulation and inflammation in autoimmune pathologies such as MS. Their diverse mechanisms of action, ranging from GPCR activation to HDAC inhibition suggest their potential as therapeutic targets. Clinical trials investigating the efficacy of SCFA supplementation in MS are warranted to translate preclinical findings into clinical practice and improve patient outcomes. By understanding the interplay between the gut microbiota by-products and the immune system, new strategies for MS therapies may emerge, focusing on modulating the gut–brain axis and restoring immune balance.

## Figures and Tables

**Table 1:** Summary of human studies regarding SCFA analysis (acetate, propionate, butyrate, valerate) and their patterns in MS patients. CIS: clinically isolated syndrome; HC: healthy controls; MS: multiple sclerosis; NMOSD: neuromyelitis optica spectrum disorder; RR: relapsing-remitting; SCFA: short-chain fatty acids; SP: secondary progressive.

Study	Sample Type	Participants	SCFA Takeaways
Levi et al. [19]	Stool samples	129 MS 58 HC	MS patients have lower levels of butyrate-producing bacteria but no difference in serum levels of butyrate between MS and controls was noted.
Saresella et al. [20]	Serum and stool samples	38 MS 38 HC	Serum levels of butyrate were significantly decreased in MS compared to controls (*p* < 0.0001) whereas caproic acid levels were statistically significantly increased (*p* < 0.0001) in MS compared to HC. The butyric acid/caproic acid ratio was significantly reduced in MS compared to HC (*p* < 0.0001)
Cuello et al. [21]	Serum samples	53 pregnant RRMS patients 21 pregnant HC	All MS patients showed significant increases in acetate levels during pregnancy and postpartum compared to HC. Higher propionate values were noted in non-active patients compared to active patients. Propionate and butyrate values were associated with relapses. Low propionate/acetate ratio in the first trimester had a higher risk of relapses during pregnancy and postpartum (*p* < 0.0001)
Park et al. [22]	Plasma samples	20 SPMS 15 HC	All SCFA levels were statistically significantly reduced in SPMS patients compared to HC (all *p* < 0.05).
Moles et al. [23]	Stool samples	20 MS 20 HC	Total SCFA fecal concentrations are statistically significantly reduced in MS compared to HC (*p* = 0.056). Based on EDSS stratification (≤1.0 and >1.0), acetate levels are significantly lower in the first group (*p* = 0.015); butyrate and caproic acid levels are significantly higher in the first group (*p* = 0.002/0.05)
Becker et al. [24]	Stool samples	41 MS 35 HC	All SCFA concentrations were descriptively reduced in RRMS compared to HC but no statistically significant differences were obtained. For the MS group, all SCFA concentrations except for valerate were statistically significantly lower in women compared to men (all *p* < 0.05).
Zeng et al. [25]	Stool samples	34 MS 34 NMOSD 34 HC	All SCFA concentrations were statistically significantly reduced in MS patients compared to HC (all *p* < 0.05). Compared to NMOSD patients, the MS patients had higher acetate and butyrate levels
Takewaki et al. [26]	Stool samples	12 RRMS 9 SPMS 8 HC	All SCFA concentrations were significantly lower in RRMS compared to HC (*p* < 0.05). The SCFA concentrations were descriptively more reduced in SPMS compared to HC.
Trend et al. [27]	Serum samples	30 CIS and MS patients 10 HC	Significantly lower levels of propionate in CIS/MS patients compared to controls (*p* = 0.0008).
Duscha et al. [28]	Serum and stool samples	268 MS patients 68 HC	Significantly lower levels of propionate in stool and serum samples in MS patients compared to controls (*p* < 0.05)
Dominguez-Mozo et al. [29]	Plasma samples	191 MS 79 HC	Statistically significant higher acetate levels were found in untreated MS patients compared to treated and HC (*p* = 0.04). Untreated MS patients exhibited lower ratios of propionate/acetate and butyrate/acetate (*p* = 0.012/0.008).
Olsson et al. [30]	Serum samples	58 MS 50 HC	Total SCFA concentration levels were reduced in MS patients compared to HC (*p* = 0.22). Acetate levels were statistically significantly lower in MS compared to HC (0.021). Ratio acetate/butyrate and acetate/(propionate + butyrate) were significantly lower in MS compared to HC (*p* = 0.005/0.01).
Pérez-Pérez et al. [31]	Plasma samples	95 MS 54 HC	Acetate levels were statistically significantly higher in MS patients compared to HC (*p* = 0.003).

## Data Availability

No new data were created or analyzed in this study. Data sharing is not applicable to this article.

## Abbreviations

AMPK	AMP-activated protein kinase
BBB	blood–brain barrier
BDNF	brain-derived neurotrophic factor
CNS	central nervous system
CoA	coenzyme A
EAE	experimental autoimmune encephalomyelitis
EDSS	expanded disability status scale
GPCR	G protein-coupled receptors
HC	healthy controls
HDAC	histone deacetylase
IFN	interferon
IL	interleukin
LPS	lipopolysaccharides
MA	methyl acetate
MCT	monocarboxylate transporters
MS	multiple sclerosis
NF-κB	nuclear factor κB
RR	relapsing remitting
SCFA	short-chain fatty acids
SP	secondary progressive
TEER	transendothelial electrical resistance
TJ	tight junctions
